# Knowledge of and beliefs about palliative care in a nationally-representative U.S. sample

**DOI:** 10.1371/journal.pone.0219074

**Published:** 2019-08-15

**Authors:** Jennifer M. Taber, Erin M. Ellis, Maija Reblin, Lee Ellington, Rebecca A. Ferrer

**Affiliations:** 1 Department of Psychological Sciences, Kent State University, Kent, Ohio, United States of America; 2 Behavioral Research Program, National Cancer Institute, Bethesda, Maryland, United States of America; 3 Department of Health Outcomes and Behavior, Moffitt Cancer Center, Tampa, Florida, United States of America; 4 College of Nursing, University of Utah, Salt Lake City, Utah, United States of America; Emory University School of Public Health, UNITED STATES

## Abstract

Palliative care aims to improve quality of life for people with serious illness and their families. One potential barrier to palliative care uptake is inaccurate knowledge and/or negative beliefs among the general population, which may inhibit early interest in, communication about, and integration of palliative care following subsequent illness diagnosis. We explored knowledge and beliefs about palliative care among the general public using nationally-representative data collected in 2018 as part of the cross-sectional Health Information National Trends Survey. Only individuals who had heard of palliative care (*n* = 1,162, *M*_*age*_ = 51.8, 64% female) were queried on knowledge and beliefs. We examined whether self-assessed level of awareness of palliative care (i.e., knowing a little vs. enough to explain it) was associated with the relative likelihood of having accurate/positive beliefs, inaccurate/negative beliefs, or responding “don’t know” to questions about palliative care. Respondents who indicated knowing a lot about palliative care had more accurate versus inaccurate knowledge than those who knew a little on only two of six items and more positive attitudes on only one of three items. In particular, respondents with greater awareness were equally likely to report that palliative care is the same as hospice and requires stopping other treatments, and equally likely to believe that palliative care means giving up and to associate palliative care with death. Those with higher awareness were less likely than those with lower awareness to respond “don’t know,” but greater awareness was not necessarily associated with having *accurate* or *positive* beliefs about palliative care as opposed to inaccurate or negative beliefs. Thus, even members of the general public who perceived themselves to know a lot about palliative care were often no less likely to report inaccurate knowledge or negative beliefs (versus accurate and positive, respectively). Findings suggest a need to improve awareness and attitudes about palliative care.

## Introduction

Palliative care refers to any care aimed at improving quality of life for people with serious illness and their families, including by managing patient pain and symptom burden and addressing psychosocial issues [1], while respecting patients’ and families’ goals and needs [2]. Palliative care was originally employed in hospice for patients near end of life [2], but hospice is only one delivery model for palliative care services. Palliative care is now recommended for all patients facing serious illness [3], ideally beginning at the time of diagnosis [1] and regardless of whether patients are also pursuing life-sustaining or “curative” treatments [2–5]. Thus, palliative care provides an “extra layer of support” ([4], p. 460) for patients and their family and informal caregivers.

There is evidence for many benefits of receiving palliative care [2, 4, 6], including improved mood and quality of life [7–9] and reduced health care costs [10]. Although palliative care services are not designed to increase longevity [11] and increased patient survival length is not an explicit goal, slight increases in patient survival length have been observed [9]. Yet, despite these multiple benefits, less than 5% of people hospitalized in the U.S. in 2015 received palliative care services [12]. Some barriers to utilization are structural, such as insufficient availability of palliative care providers and programs [4, 13, 14]. However, as availability of palliative care programs has dramatically increased over the past two decades [12] and palliative care uptake remains suboptimal, it is likely that psychosocial barriers also affect uptake.

### Misperceptions and negative beliefs about palliative care among the general public

One potential psychosocial barrier to palliative care uptake early in the illness trajectory is misperceptions and negative beliefs about palliative care that caregivers or surrogate decision makers hold *before engaging with the medical system* or at the *time of diagnosis* [15–18]. Patients and family members who are not knowledgeable about palliative care *at the time of diagnosis* are unlikely to ask for a palliative consultation because they do not know to ask. Patients and family members who have negative attitudes towards palliative care *at the time of diagnosis* may be similarly unlikely to ask for a consultation and less likely to accept a consultation or services if offered [17]. These pre-existing beliefs can influence formal directives, such as advance care plans, and affect the care options available to patients by shaping provider decision making and patient-provider conversations [18–21]. For example, if a new cancer patient tells her oncologist she wants to exclusively pursue life-extending treatments because she equates palliative care with death, her oncologist may be more likely to prescribe treatments that are off-protocol or have low probability of efficacy and be less likely to discuss palliative care, even at end of life [22].

It is also essential that caregivers have accurate knowledge of palliative care [23]. Nearly one-third of U.S. adults rely on others to make important decisions regarding their end-of-life care, and most patients in the U.S. want their family to be involved in decision making [24]. Thus, it is important to understand knowledge and beliefs about palliative care among younger and healthier adults who may serve as formal or informal decision makers now or in the future. These adults may also become sick themselves and use this prior knowledge to make consequential treatment decisions.

It is important to understand the beliefs and knowledge about palliative care among the general public—those who are not yet facing diagnosis or caregiving, but who are likely to experience either or both in their lifetimes—to develop targeted interventions which promote enrollment in palliative care services. Interventions targeting patients and family members at or after the time of diagnosis may be too late: the emotional nature of a serious illness diagnosis, and of considering palliative care itself, may interfere with accurate and careful processing of new information about palliative care for those unfamiliar with the services [25].

### What is the nature of misperceptions and negative beliefs about palliative care?

Social sciences research indicates widespread misinformation, negative beliefs and attitudes, and lack of awareness about palliative care. Some studies, including one using the same dataset used in the present paper, suggest that between 50% to over 70% of adults have never heard of palliative care in both the U.S. [26–31] and other countries [32]. Even those who have heard of palliative care hold misperceptions and negative attitudes about palliative care, despite tending to know that these services aim to relieve pain and other side effects, provide comfort and dignity, and address other psychological issues [26, 28, 31, 33, 34]. People often associate palliative care with death and end-of-life care [15, 31, 34, 35]. One misperception is that palliative care involves forgoing life-sustaining care or other treatments [28, 31], even though palliative care is recommended regardless of whether a patient is pursuing other treatments [3]. For example, in one U.S. study, nearly three-quarters of those who had heard of palliative care associated it with end-of-life care and less than 10% mentioned that it could be employed at any point in the illness [26]. Similarly, people believe that hospice care is “only about death” or is equivalent to “giving up” [36]. As palliative care is often conflated with hospice, and awareness of hospice is higher than awareness of palliative care [26], these negative beliefs about hospice might extend to palliative care. Social (mis)perceptions about morphine may also cause patients to overestimate likelihood of addiction and death, leading them to fear these medications and avoid palliative care services, such as pain management [37, 38].

### The current study

Much of the prior research on the public’s knowledge and attitudes about palliative care has occurred outside of the U.S. [15, 34, 35]. Because countries differ in their implementation of palliative care–for example, the U.K. has more hospital-based hospices than in the U.S. where the predominant hospice model is home-based care [39] (but see [40])–knowledge and beliefs held in one country may not generalize to another. Moreover, as previously noted, the availability of palliative care services has dramatically increased in the last two decades [14]. In this rapidly evolving landscape, it is important to monitor changes in the public’s awareness, knowledge, and attitudes about palliative care. Recently, researchers have reported beliefs about palliative care among individuals in the U.S. who rated their own knowledge of palliative care as higher versus lower among the same sample used here [31]. Overconfident individuals are of interest because if people inaccurately perceive themselves to be knowledgeable about palliative care, they may be unmotivated to seek corrective information when they or a loved one is diagnosed with an illness, and they may spread inaccurate information to others. We built upon these prior analyses by also examining the likelihood of responding “don’t know” to palliative care items instead of providing a response on the continuum from agree to disagree. We sought to understand people who responded “don’t know” because these people who respond with “don’t know” are unique socio-demographically and with respect to their health behaviors. For example, don’t know responders report greater avoidance of health information, lower health-related self-efficacy, and lower education levels [41–46]. They are also less likely to engage in health-promoting behaviors, even after adjusting for demographic risk factors [47–48]. This growing body of evidence suggests that responding don’t know is not equivalent to skipping the item and that excluding these responses as missing data may be masking conceptually meaningful differences between don’t know and other responders. Moreover, by including don’t know responders in the current set of analyses, we extended our ability to examine differences between those who are knowingly versus unknowingly uninformed or misinformed about palliative care.

A secondary aim was to examine whether a set of sociodemographic factors was associated with palliative care knowledge and beliefs: gender, age, education, race/ethnicity, and personal and familial history of cancer. We hypothesized that women would have more accurate knowledge and more positive attitudes than men based on prior research in which women reported greater awareness [32], higher knowledge [28, 34], less likelihood of recommending to others [26], and lower uptake [49] of palliative care. We similarly expected older adults and those with higher levels of education to have greater knowledge of palliative care based on prior research [28, 34], with no predictions for positivity of beliefs. We hypothesized that relative to White participants, Black and Hispanic or Latinx participants would have less positive beliefs about palliative care [50], with no predictions for knowledge. Finally, we predicted that individuals with a personal or family history of cancer would have greater knowledge and more positive beliefs, as these individuals may have experience with palliative care, and greater awareness and understanding of palliative care has been associated with more positive attitudes [26, 29].

## Methods

### Study design and population

Data were collected from a mailed survey administered from January to May 2018 as part of the Health Information National Trends Study (HINTS) 5, Cycle 2. HINTS is a cross-sectional, nationally representative U.S. survey that targets adults aged 18 or older in the civilian non-institutionalized population. HINTS assesses the need for, access to, and use of health-related information and health-related behaviors, perceptions, and knowledge [51]. The survey was completed by 3,504 participants, with a household response rate of 32.4%. Full details of the study design are available at hints.cancer.gov. HINTS was approved by the National Institutes of Health Special Studies Institutional Review Board.

### Measures

All respondents answered the item, “How would you describe your level of knowledge about palliative care?” Those who indicated “I’ve never heard of it” (*n* = 2,283, 70.2% of the sample, weighted) were not asked follow-up questions to assess knowledge of and beliefs about palliative care. Participants who selected one of the other two responses (*n* = 1,162; 28.4% of the sample, weighted)—“I know a little bit about palliative care” or “I know what palliative care is and could explain it to someone”—answered follow-up questions.

#### Palliative care knowledge and beliefs

Four items assessed perceptions of the goals of palliative care and five items assessed additional perceptions about palliative care. Some items were adapted from prior research [29] and all items underwent pretesting and cognitive interviewing to ensure they were interpreted as intended. All nine items were analyzed separately and had response options of 1 = strongly agree, 2 = somewhat agree, 3 = somewhat disagree, and 4 = strongly disagree, and “don’t know.” Each goal item began with, “To me, the goal of palliative care is…” and the items were: 1) Help friends and family to cope with a patient’s illness; 2) Offer social and emotional support; 3) Manage pain and other physical symptoms; and 4) Give patients more time at the end of life.

An additional five items assessed beliefs about palliative care: “Accepting palliative care means giving up;” “It is a doctor’s obligation to inform all patients with cancer about the option of palliative care;” “If you accept palliative care, you must stop other treatments;” “Palliative care is the same as hospice care;” and “When I think of ‘palliative care,’ I automatically think of death.”

We considered six of these nine items to have an objectively correct answer based on current evidence and conceptualized them as “knowledge” items. Knowledge items and their correct responses are shown in Table 1. Of note, the item that assessed whether a goal of palliative care is to extend length of life was considered to be objectively *inaccurate* because life-extension is not an explicit goal of palliative care despite evidence that some specific palliative care interventions have briefly delayed mortality [9] (but see [6]). (Of note, this item was originally developed to examine the prevalence of an inaccurate belief about palliative care. However, we recognized that endorsement of this item also constitutes a positive belief about palliative care, in contrast with other belief items in which inaccurate beliefs also reflect negative attitudes.) The remaining three items were considered to not have evidence-based accurate or inaccurate responses and were thus conceptualized as either positive or negative “beliefs” about palliative care (Table 1).

**Table 1:** Knowledge and beliefs about palliative care means, standard errors, and frequencies of specific responses.

	Mean (SE)^1^	Agree, %	Disagree, %	Don’t know, %
**Knowledge of palliative care**				
The goal of palliative care is to help friends and family to cope with a patient’s illness. (*n* = 1,122)	3.47 (0.03)	**90.6**	6.2	3.2
The goal of palliative care is to manage pain and other physical symptoms. (*n* = 1,129)	3.75 (0.02)	**95.1**	1.8	3.1
The goal of palliative care is to offer social and emotional support. (*n* = 1,115)	3.58 (0.02)	**93.4**	3.4	3.2
The goal of palliative care is to give patients more time at the end of life. (*n* = 1,109)	2.87 (0.04)	59.3	**32.3**	8.5
Palliative care is the same as hospice care. (*n* = 1,129)	2.13 (0.05)	31.7	**53.0**	15.3
If you accept palliative care, you must stop other treatments. (*n* = 1,133)	1.67 (0.04)	14.5	**70.4**	15.1
**Beliefs about palliative care**				
Accepting palliative care means giving up. (*n* = 1,127)	1.60 (0.03)	15.1	**81.3**	3.6
When I think of "palliative care," I automatically think of death. (*n* = 1,135)	2.23 (0.04)	42.5	**53.7**	3.8
It is a doctor’s obligation to inform all patients with cancer about the option of palliative care. (*n* = 1,136)	3.43 (0.04)	**83.3**	9.6	7.1

^1^ Response options ranged from 1–4. Higher scores indicate greater agreement.

*Notes*. Bold type indicates accurate knowledge and positive beliefs. Sample sizes are lower for means than frequencies because the continuous coding excluded “don’t know” responses.

#### Demographic and medical factors

All demographic and medical factors were self-reported. Participants indicated their age in years and sex (Female = 0, Male = 1). Level of educational attainment was treated as continuous, ranging from 1 = less than 8 years to 7 = postgraduate. Race was categorized using two separate variables: 1) 1 = White, 0 = non-White, and 2) 1 = Black or African American, 0 = not Black or African American. Ethnicity was categorized as 1 = Hispanic or 0 = non-Hispanic. Racial/ethnic categories were not mutually exclusive.

Personal history of cancer was assessed with the item, “Have you ever been diagnosed as having cancer?” Family history of cancer was assessed with “Have any of your family members ever had cancer?” For both items, responses were coded as 1 = Yes or 0 = No or Not Sure.

### Overview of analyses

Analyses were conducted among the 1,162 participants who indicated having heard of palliative care. Before the data were released, missing values for age, gender, educational attainment, marital status, race, and ethnicity were replaced with the value reported by a similar case using hot-deck imputation. The exact sample size differs across analyses due to missing data on individual knowledge and belief items. To account for the complex sampling design, a set of 50 jackknife replicate weights was used to generate nationally representative parameter estimates [51]. All analyses were conducted using SAS-callable SUDAAN version 11.0.1 and Stata version 15. All frequencies are unweighted and all percentages and inferential statistics are weighted. The significance level was set at *p* < .05.

The palliative care knowledge and belief items were scored in two ways. First, we reverse scored items so higher values indicated greater agreement then calculated mean scores ranging from 1 to 4, with “don’t know” responses treated as missing. Second, we created 3-level variables of agreement (1 = strongly agree and somewhat agree) vs. disagreement (2 = strongly disagree and somewhat disagree”) vs. “don’t know” (0 = don’t know).

We first examined demographic and medical history characteristics of participants who had heard of palliative care. We next examined zero-order correlations among knowledge and belief items using the continuous scoring. Next, we examined the weighted proportions of individuals with accurate knowledge, positive beliefs, and don’t know (DK) responses for all knowledge and belief items.

We then conducted a series of weighted multinomial logistic regressions to examine whether the level of self-assessed awareness (hereafter referred to as awareness) was associated with the likelihood of responding agree, disagree, or DK to the nine items, controlling for demographic and medical factors (i.e., age, gender, education, race, ethnicity, and personal and family history of cancer). These regressions used the 3-level variable as the outcome, allowing us to separately compare DK responders to 1) those with accurate knowledge and positive beliefs and 2) those with inaccurate/negative responses. Analyzing DK responders as their own group is important because inaccurate/negative and DK responses may not be conceptually equivalent. We also compared those with accurate/positive responses to those with inaccurate/negative responses.

We then used *t*-tests and chi-square tests to examine bivariate associations between the degree of awareness about palliative care and the seven aforementioned demographic and medical factors. Finally, a series of multinomial logistic regression models was used to examine whether the likelihood of responding agree, disagree, or DK to the nine items was predicted by the seven demographic and medical factors when entered simultaneously.

## Results

### Participant characteristics

Among the 1,162 participants who had heard of palliative care, the average age was 51.8 years (*SE* = 0.60). Most identified as White (82.2%, *n* = 902), with a subset identifying as Black or African American (10.1%, *n* = 127). A minority identified as Hispanic ethnicity (8.7%; *n* = 97). Nearly two-thirds (64.0%, *n* = 806) were female (*n* = 348 males). Most reported having obtained a college degree or higher (46.5%, *n* = 702) versus having completed some college (40.3%, *n* = 312) or completed high school or less (13.3%, *n* = 135). Most had a family history of cancer (76.9%, *n* = 910; *n* = 237 did not have a family history) and did *not* have a personal history of cancer (88.8%, *n* = 954; *n* = 207 had a personal history).

### Associations among palliative care knowledge and belief items

Correlations among palliative care knowledge and belief items are shown in Table 2. All four items assessing perceived goals of palliative care were significantly positively correlated. The highest correlation was among knowledge that a goal of palliative care is to help friends and family cope and to offer social and emotional support (*r* = 0.65). No other individual correlations were above *r* = 0.40, suggesting that items assessed distinct but related constructs. Reporting that palliative care is the same as hospice care was significantly correlated with more negative beliefs about palliative care for two of the three items—accepting palliative care means giving up and associating palliative care with death—and with inaccurately expressing that accepting palliative care involves stopping other treatments.

**Table 2:** Correlations among knowledge and beliefs about palliative care.

	1	2	3	4	5	6	7	8
**Knowledge of palliative care**								
1. The goal of palliative care is to help friends and family to cope with a patient’s illness.	--							
2. The goal of palliative care is to manage pain and other physical symptoms.	0.24**	--						
3. The goal of palliative care is to offer social and emotional support.	0.65**	0.37**	--					
4. The goal of palliative care is to give patients more time at the end of life.	0.34**	0.12**	0.28**	--				
5. Palliative care is the same as hospice care.	0.01	0.00	0.02	0.15*	--			
6. If you accept palliative care, you must stop other treatments.	-0.06	0.02	-0.12	-0.00	0.35**	--		
**Beliefs about palliative care**								
7. Accepting palliative care means giving up.	-0.11	-0.05	-0.17*	-0.01	0.27**	0.35**	--	
8. When I think of "palliative care," I automatically think of death.	-0.11	-0.02	-0.16*	-0.09	0.40**	0.29**	0.33**	--
9. It is a doctor’s obligation to inform all patients with cancer about the option of palliative care.	0.05	0.13*	0.06	0.13*	0.09	0.04	0.00	0.04

*p < .05

**p < .001

### Proportion of accurate knowledge, positive beliefs, and DK responses about palliative care

The weighted proportions of respondents that agreed, disagreed, and responded DK for individual items are shown in Table 1.

#### Knowledge about palliative care

Most respondents (>90%) were aware that goals of palliative care include managing pain and symptoms, helping friends and family cope, and providing social and emotional support. Slightly over two-thirds (70.4%) of respondents were aware that if you start palliative care you do not need to stop other treatments. However, only half (53.0%) were aware that palliative care and hospice are not the same and less than one-third (32.3%) were aware that giving patients more time is *not* an explicit goal of palliative care.

#### Beliefs about palliative care

As shown in Table 1, most respondents (81.3%) expressed the positive belief that palliative care does not mean giving up and over 80% believed it was a doctor’s obligation to inform cancer patients about palliative care. However, 42.5% expressed the negative belief that they automatically associate palliative care with death.

#### Don’t know responding

The highest rates of DK responses were for the knowledge items that assessed whether palliative care was the same as hospice care and whether accepting palliative care means stopping other treatments (Table 1). For both, over 15% of respondents responded DK. The proportion of respondents who responded DK for other items ranged from 3.1% to 8.5%.

### Knowledge and beliefs as a function of awareness of palliative care

When indicating their degree of awareness about palliative care, the majority (62.4% *n* = 712) expressed lower awareness: “I know a little bit about palliative care” (17.9% of entire HINTS sample). One-third (37.6%, *n* = 450) expressed greater awareness: “I know what palliative care is and could explain it to someone” (10.8% of entire HINTS sample).

**Fig 1:**
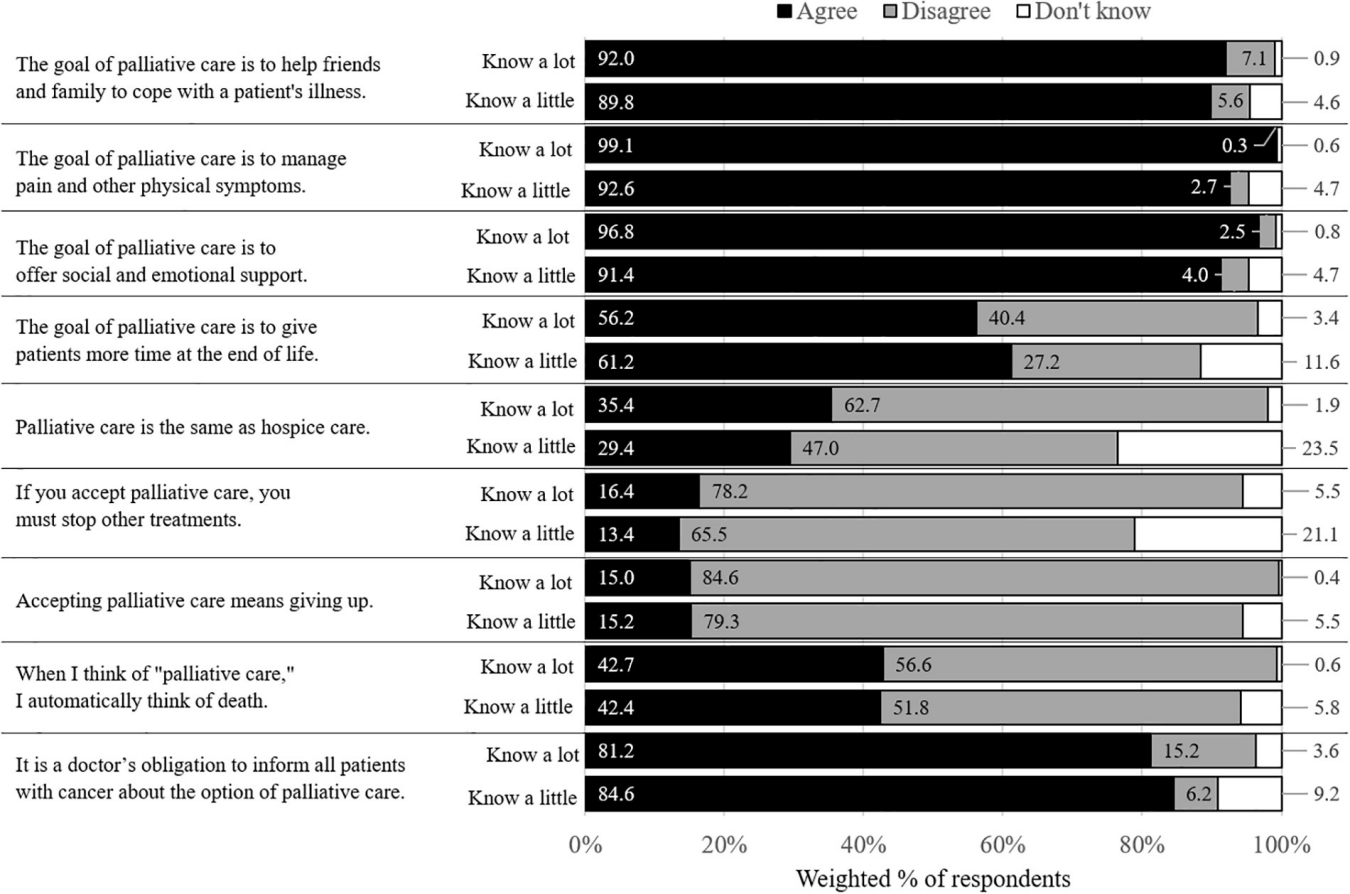
Proportion of respondents agreeing, disagreeing, and responding DK with each palliative care knowledge and belief item as a function of self-assessed awareness about palliative care (know a lot versus know a little).

The weighted proportions of respondents agreeing, disagreeing, and responding DK to each knowledge item as a function of awareness of palliative care are shown in Fig 1. Among those with greater awareness of palliative care, slightly over one-third (35.4%; *n* = 157) expressed the inaccurate belief that palliative care is the same as hospice and 16.4%; (*n* = 68) expressed the inaccurate belief that palliative care means stopping other treatment. Further, 56.2% (*n* = 244) of those with greater awareness expressed the belief that a goal of palliative care is to give patients more time. As a reminder, we considered this response to be objectively inaccurate, but it also represented positive beliefs about palliative care. Additionally, among those with higher awareness, negative beliefs ranged from 15.0% (*n* = 62) for the belief that accepting palliative care means giving up to 42.7% (*n* = 174) for associating palliative care with death (Fig 1).

#### Knowledge about palliative care as a function of awareness

We next conducted multinomial logistic regressions to assess whether degree of awareness of palliative care (knowing a little versus knowing a lot) was associated with knowledge and beliefs about palliative care. Compared to those with lower awareness of palliative care, participants with greater awareness were less likely to respond with DK, but they did *not* consistently demonstrate more accurate knowledge (Table 3). Specifically, for all knowledge items, participants with greater awareness were more likely to have accurate knowledge than to respond DK compared to those with lower awareness (this is indicated by agreement with the first three knowledge items in Table 3 and by disagreement with the last three knowledge items in Table 3). However, participants with higher versus lower awareness were *also* more likely to have *inaccurate* knowledge than to respond DK for four of the six knowledge items (i.e., that goals of palliative care are to help friends and family cope and to extend life, that palliative care is the same as hospice, and that accepting palliative care means stopping other treatments; Table 3). Further, relative to respondents with lower awareness, greater awareness was associated with higher likelihood of having accurate versus inaccurate knowledge for only two of the six knowledge items: that goals of palliative care include managing symptoms but not life-extension.

**Table 3:** Multinomial regressions with the degree of self-assessed awareness (know a lot versus know a little) about palliative care predicting the likelihood of a) agreeing versus disagreeing, b) agreeing versus responding “don’t know,” and c) disagreeing versus responding “don’t know” for knowledge and belief items.

	Agree vs. Disagree (ref)		Agree vs. Don’t know (ref)		Disagree vs. Don’t know (ref)	
	RRR	*p* value	RRR	*p* value	RRR	*p* value
**Knowledge of palliative care**						
The goal of palliative care is to help friends and family to cope with a patient’s illness.	0.81	.591	**5.11**	**.001**	6.29	.003
The goal of palliative care is to manage pain and other physical symptoms.	8.45	.001	**9.28**	**.001**	1.10	.919
The goal of palliative care is to offer social and emotional support.	1.71	.336	**6.22**	**.002**	3.63	.104
The goal of palliative care is to give patients more time at the end of life.	0.62	.016	3.15	.002	**5.08**	**< .001**
Palliative care is the same as hospice care.	0.90	.611	14.82	< .001	**16.39**	**< .001**
If you accept palliative care, you must stop other treatments.	1.03	.925	4.71	< .001	**4.59**	**< .001**
**Beliefs about palliative care**						
Accepting palliative care means giving up.	0.93	.765	15.70	.002	**16.94**	**.001**
When I think of "palliative care," I automatically think of death.	0.92	.669	9.25	< .001	**10.03**	**< .001**
It is a doctor’s obligation to inform all patients with cancer about the option of palliative care.	0.39	.005	**2.48**	**.037**	6.34	.001

*Notes*. RRR = relative risk ratio. Bold type indicates accurate knowledge and positive beliefs. Analyses controlled for demographic and medical factors (i.e., age, gender, education, race, ethnicity, and personal and family history of cancer).

#### Beliefs about palliative care as a function of awareness

Similar to the results for knowledge, respondents with greater awareness of palliative care typically were less likely to respond DK but did not have more positive beliefs than those with lower awareness (Table 3). For all belief items, participants with greater awareness were more likely to have positive beliefs than to respond DK compared to those with lower awareness (as indicated by disagreement with the first two belief items in Table 3 and by agreement with the last belief item in Table 3). However, participants with higher versus lower awareness were *also* more likely to have *negative* beliefs than to respond DK for all three belief items. Relative to respondents with lower awareness, those with higher awareness were more likely to express positive versus negative beliefs for only one of three items: that doctors are obligated to inform patients about palliative care.

### Participant characteristics as predictors of knowledge and beliefs

We used chi-square tests to examine whether awareness about palliative care differed as a function of demographic and medical factors (i.e., age, gender, education, race, ethnicity, and personal and family history of cancer; see also [27, 31] for demographic characteristics of those with no, low, and high awareness). The proportion of individuals with higher awareness differed only as a function of educational attainment and personal cancer history. Specifically, those who expressed greater awareness had more years of education (*M* = 3.45, SE = 0.04, where 3 = “12 years” and 4 = “post high school training other than college”) than those who expressed lower awareness (*M* = 3.22, *SE* = 0.05) and a higher proportion of participants with a personal history of cancer expressed greater awareness (48.3%) compared to those without a cancer history (36.3%; *χ*^*2*^(1) = 4.80, *p* = .033). There were no differences in awareness as a function of age (*t*(49) = 0.59, *p* = .561), gender (*χ*^*2*^(1) = 3.69, *p* = .060), White race (*χ*^*2*^(1) = 2.31, *p* = 0.135), Black race (*χ*^*2*^(1) = 0.17, *p* = 0.680), Hispanic ethnicity (*χ*^*2*^(1) = 0.28, *p* = 0.598), or family history of cancer (*χ*^*2*^(1) = 0.07, *p* = 0.795).

#### Demographic and medical factors associated with knowledge about palliative care

Finally, we used multinomial logistic regression to examine whether demographic and medical factors were associated with responses to the nine palliative care knowledge and belief items when entered simultaneously. Similar to analyses conducted with awareness as a predictor, analyses were conducted to test whether each of these factors influenced the likelihood of 1) agreeing versus disagreeing, 2) agreeing versus responding “don’t know,” and 3) disagreeing versus responding “don’t know.” Complete results are shown in S1 Table and all significant predictors are reported in the following sections.

Level of educational attainment was associated with more accurate knowledge for all six items. Specifically, relative to those with fewer years of educational attainment, those with more years of educational attainment were 1) more likely to disagree (versus agree or respond DK) that a goal of palliative care is to give patients more time, that palliative care is the same as hospice care and that palliative care means stopping other treatments, and 2) more likely to agree (versus respond DK) that a goal of palliative care is to offer support. However, those with greater education were also more likely to agree *and* disagree (versus respond DK) that goals of palliative care are to help friends and family cope and to manage symptoms.

Age was associated with all four items assessing knowledge of goals but not with the other two knowledge items. Contrary to our hypotheses, younger adults typically reported more accuracy and were less likely to respond DK: relative to older adults, they were 1) more likely to agree (versus disagree and respond DK) that a goal is to offer support, 2) more likely to agree (versus disagree) that a goal is to manage symptoms, 3) more likely to agree (versus respond DK) that a goal of palliative care is to help friends and family cope, but 4) more likely to agree *and* disagree (versus respond DK) that a goal is to extend life.

Relative to non-White adults, White adults had greater knowledge for two of the six items: they were more likely to agree (versus respond DK) that a goal is to help family and friends cope and more likely to agree (versus disagree and respond DK) that a goal is to offer support. Black adults were more likely to agree (versus disagree) that a goal of palliative care is to offer support. Hispanic ethnicity was not associated with responses to any of the knowledge items. Those with a personal history of cancer were more likely agree *and* disagree (versus respond DK) that a goal of palliative care is to extend life; those with a family history of cancer were more likely to agree (versus disagree) that a goal of palliative care is to offer support. Contrary to our hypotheses, gender was not associated with responses to any of the knowledge items.

#### Demographic and medical factors associated with beliefs about palliative care

Level of educational attainment was associated with less likelihood of responding DK, and sometimes more positive beliefs, for all three items. Specifically, relative to those with fewer years of educational attainment, those with more years of educational attainment were 1) more likely to agree (versus respond DK) that doctors are obligated to inform patients about palliative care; 2) more likely to disagree (versus agree) and also more likely to agree *and* disagree (versus respond DK) that accepting palliative care means giving up, and 3) more likely to agree *and* disagree (versus respond DK) that they associated palliative care with death.

Younger adults were less likely to respond DK for two of the three items; specifically, compared to older adults, they were more likely to agree and disagree (versus respond DK) that accepting palliative care means giving up and that they associate palliative care with death. Hispanic adults were more likely to respond DK (versus disagree) that accepting palliative care means giving up and more likely to disagree (versus agree) that doctors have an obligation to inform patients about palliative care. White adults were more likely to disagree (versus agree and respond DK) that accepting palliative care means giving up whereas Black adults were more likely to disagree (versus agree) that palliative care means giving up. Personal and family history of cancer and gender were not associated with responses to any of the belief items.

## Discussion

In a nationally representative sample of U.S. adults, nearly three-quarters (71.6%) had never heard of palliative care [27]. Consistent with similar analyses of these and other data, among those who had heard of palliative care, between 90–95% expressed accurate knowledge that the goals of palliative care include managing pain and other physical symptoms, offering social and emotional support, and helping friends and family cope [26, 28, 31, 33]. Further, accuracy was lower for other aspects of palliative care knowledge: about one-third endorsed the inaccurate belief that palliative care is the same as hospice care, and one-third either agreed with or did not know whether accepting palliative care requires stopping other treatments (see also [31]). Negative beliefs about palliative care were common: in particular, over 40% of participants associated palliative care with death (see also [31]).

Even individuals who indicated that they could explain palliative care to someone else, held inaccurate knowledge and negative beliefs. Those with higher awareness were less likely than those with lower awareness to respond “don’t know,” but similarly likely to respond with either agree or disagree for the majority of knowledge and belief items. In other words, greater awareness did not necessarily result in people having *accurate* or *positive* information versus inaccurate or negative information about palliative care. This pattern is particularly striking for beliefs about whether palliative care is the same as hospice: only 2% of those with high awareness responded DK versus 25% of those with lower awareness. Instead of responding DK, respondents with higher awareness were more likely to *both* accurately reject this statement as false (63% vs. 47%) *and* inaccurately accept this statement as true (35% vs. 29%) compared to those with lower awareness. In the present study, DK responses were retained as a distinct category, given research indicating that DK responders have distinct characteristics such as lower health-related self-efficacy and lower educational attainment [41, 42]. Treating DK responses as missing data or combining them with inaccurate and negative belief responses would have misrepresented the data by indicating exaggerated differences in knowledge and beliefs as a function of awareness. From a methodology perspective, it is important to understand characteristics associated with and reasons underlying DK responding to inform the design of surveys that minimize DK responding.

From a research perspective, the findings demonstrating that many respondents were overconfident in their knowledge of palliative care suggest that it would be insufficient to merely ask individuals if they understand palliative care and to assume that affirmative responses indicate accurate knowledge. Because people might overstate their understanding to avoid appearing uninformed, researchers might obtain better data if they define palliative care before eliciting knowledge and attitudes.

We were unable to differentiate among respondents who had been diagnosed with a serious illness versus those who had not, and it is possible that people with serious illness and their caregivers were more likely to be aware of palliative care and had accurate knowledge and positive beliefs. However, fewer than half of respondents with a personal history of cancer had heard of palliative care, and neither personal nor family history of cancer was associated with 4 of the 6 knowledge items or any of the belief items. Thus, given that some patients with serious illness are likely to be either unaware or misinformed about palliative care, medical providers might consider initiating conversations about palliative care before patients broach the subject. The vast majority of U.S. adults have either not heard of palliative care before becoming ill (and thus do not know to ask for it), or–and perhaps more problematically–they know about palliative care but are unaware that their information needs correction. That people’s misperceptions were usually negatively-biased reduces the likelihood that patients will request palliative care. Both possibilities require provider-initiated correction, ideally aimed at both patients and their caregivers. These data also indicate two groups who may require different interventions: one group is uninformed and the other group is misinformed (and unaware of it).

Over half of respondents endorsed that a goal of palliative care is to extend length of life, with nearly twice as many endorsing this goal than did not. Endorsing this goal likely represents positive attitudes towards palliative care, given Western medicine’s focus on living longer. Although this belief is inconsistent with meta-analytic evidence [6] and stated goals of palliative care [11], some individuals may endorse it not necessarily because they are misinformed, but because they have heard about research showing that palliative care has extended length of life [9]. For example, Temel and colleagues’ seminal findings that palliative care slightly increased longevity among patients with metastatic non-small-cell lung cancer patients study was highlighted in Atul Gawande’s bestselling *Being Mortal* [52]. Clinicians may also use this and other studies as evidence that palliative care is not “giving up.” Future research is needed to determine the effects of intentionally or unintentionally blurring the distinctions between the goals of palliative care as opposed to life-sustaining treatments. Although such an approach may increase the acceptability of palliative care among patients, there may be unintended consequences related to patients’ and caregivers’ prognostic understanding and knowledge of their disease and treatment. For example, people who know that extending life is not an explicit goal of palliative care might have more realistic expectations about what palliative care can accomplish. A methodological limitation of the data collection may also have resulted in over-endorsement of this belief: the three preceding goal items were all accurate statements, and respondents may have assumed/inferred that this statement was also accurate.

Interestingly, reporting accurate knowledge about multiple goals of palliative care was not associated with whether individuals associated palliative care with death. Thus, being knowledgeable about the goals of palliative care did not necessarily attenuate the stigma surrounding it. This suggests that educational efforts that improve knowledge alone are unlikely to automatically improve attitudes. Of note, this runs somewhat counter to prior research in which greater awareness and positive beliefs co-occurred: individuals who knew what palliative care was or who were provided definitions tended to have more positive attitudes towards palliative care [26]. In a study with New York adults, after being provided with a definition, over 90% said they would recommend palliative care to a loved one who had a serious illness [26]. Future research is needed to determine the extent to which increasing knowledge of palliative care would increase its uptake if knowledge is not accompanied by reduced stigma.

### What are the sources of inaccurate knowledge and negative beliefs, and how can they be reduced?

Most U.S. adults have never heard the term “palliative care” [27]. Awareness and knowledge of palliative care may be hindered by social norms around talking openly about death and dying, including a reluctance among the general public to talk about death and dying [34]. Ongoing programs such as Death Over Dinner (https://deathoverdinner.org/) and Before I Die, (https://beforeidieproject.com/)–alongside attempts to ensure the transmission of accurate information—may bring more attention to this topic and reduce death-related stigma.

Among those who are aware of palliative care, more research is needed to identify sources of misperceptions and negative beliefs, such as medical providers, informal lay networks, and media. Among U.S. adults who have heard of palliative care, nearly 80% reported that health care providers would be the most trusted source to seek information about palliative care and 55% said providers would be the first source [31]. These data suggest that providers may be one source of misperceptions. Reported satisfaction with palliative care services is high [53], but some patients have cited medical providers as the source of their misperceptions [35], and many providers have low levels of awareness and inaccurate knowledge [4, 54–57]. Providers sometimes describe palliative care as a last resort or an alternative to other treatment [35], and often introduce palliative care “near the end of the patients’ journey” (p. 5) rather than at diagnosis or when treatment begins [34]. Additionally, providers who frame palliative care as pain management could cause concerns about opioid addiction among patients [37, 38]. Additionally, 30.5% of U.S. adults reported that they would first turn to the internet and social media to obtain information about palliative care [31], and over 80% of the U.S. population reported that they first went to the internet the most recent time they looked for information about health or medical topics [58]. Thus, another likely source of misperceptions about palliative care is the internet.

Another possible contributor to negative beliefs about palliative care is the label itself. In one study, medical providers were more likely to associate the term palliative care with hospice and end of life (57%) than the term supportive care (15%) [59]. These providers rated themselves more likely to prefer services with the name supportive care for patients at earlier disease stages, but preferred services with the name palliative care for patients at later disease stages [60]. Patients who had been through palliative care disliked that the term palliative care was associated with end-of-life care but had mixed views about whether the label “supportive care” was a better label [35]. This labeling issue is also a limitation of the current study: some people may only know palliative care by the terms supportive or comfort care. As the field of palliative care progresses, a consistent label may facilitate awareness and educational efforts [59].

In the present study, respondents who conflated palliative with hospice care were also more likely to associate palliative care with death and to think that palliative care means stopping other treatments and giving up. Although these data are not causal, they suggest that an important goal of educational attempts may be to distinguish that hospice is only one model through which palliative care is provided and distinct from typical inpatient or outpatient palliative care services. This may correct misperceptions that palliative care and hospice are the same, but also reduce the association of the term palliative care with end of life and death. Indeed, associating palliative care with death may serve as an emotional barrier deterring patients (and their caregivers) from these services.

Educational efforts should target awareness, knowledge, and beliefs about palliative care, and our results provide direction as to who to target. Targeting populations with low levels of education may be a key place to start, as education was the only demographic factor associated with all palliative care knowledge and belief items. Although speculative, this association could be a function of those with lower education having less access to medical care and/or lower health literacy, and thus less accurate knowledge. Contrary to hypotheses that older adults would have greater knowledge of palliative care [28, 34], younger adults reported more accurate knowledge of goals and were less likely to respond DK than older adults. This finding could be indicative that efforts to increase awareness of palliative care are succeeding. Contrary to prior research, gender was unassociated with knowledge and beliefs. We also found limited evidence of consistent knowledge or belief differences as a function of race or ethnicity.

### Limitations

One limitation of the present study is the demarcation between knowledge and beliefs. Prior researchers have considered beliefs to be “verifiable facts” [61, 62] but we conceptualized “verifiable facts” as indicating knowledge and beliefs as items that did not have correct or incorrect answers. Although we labeled “accepting palliative care means giving up” as a belief, others might consider this statement to be objectively inaccurate. Additionally, the general public is unlikely to be capable of disentangling their knowledge from their beliefs. A related limitation is that the palliative care knowledge and belief items were not from a validated scale, although some items were adapted from a previous source [29] and all items underwent pretesting and cognitive interviewing. Additionally, many of the knowledge items had limited variability in that the majority of respondents who had heard of palliative care demonstrated accurate knowledge. A future research goal may be to create a scale that assesses multiple components of palliative care knowledge and beliefs and that is better able to distinguish among people with varying degrees of knowledge. Due to the large number of analyses conducted, which increases the possibility of Type I error, we report but emphasize caution interpreting results of the analyses with demographic and medical factors as predictors of palliative care knowledge and beliefs.

### Conclusion

Palliative care programs are relatively new, and thus awareness among the general public was low and misperceptions were common. Similar to prior research [31], we found that many adults have inaccurate knowledge and negative beliefs about palliative care, but we built upon this prior work by examining how self-assessed awareness about palliative care was associated with accurate versus inaccurate beliefs versus stating “don’t know.” The present data provide a baseline to examine changes in knowledge and beliefs over time. Ultimately, interventions will need to do more than just improve knowledge and attitudes, as knowledge and attitudes alone are typically insufficient to promote behavior change [63]. Ideally, individuals would learn about palliative care programs before ever being in a situation requiring treatment decisions to be made. Increasing knowledge and positive beliefs about palliative care should lead to more informed decision making about whether and when to obtain palliative care, and future research should examine how individuals’ preexisting misperceptions and beliefs shape care trajectories.

## Supporting information

S1 Table Relative risk ratios and significance values for demographic and medical history factors entered as simultaneous predictors of accuracy and positivity of palliative care knowledge and beliefs. (DOCX)
