# Supplementary material for: Knowledge of and beliefs about palliative care in a nationally-representative U.S. sample
Source: PLoS One. 2019 Aug 15;14(8):e0219074. doi: 10.1371/journal.pone.0219074 (PMC6695129; doi:10.1371/journal.pone.0219074)
Supplement: S1 Table — (DOCX) [file pone.0219074.s001.docx]

**Supporting Information**

**S1 Table. Relative risk ratios and significance values for demographic and medical history factors entered as simultaneous predictors of accuracy and positivity of palliative care knowledge and beliefs.**

|  | Agree vs. Disagree (Ref) | Agree vs. Don’t know (Ref) | Disagree vs. Don’t know (Ref) |
| --- | --- | --- | --- |
| **Knowledge about palliative care** |  |  |  |
| The goal of palliative care is to help friends and family to cope with a patient's illness. |  |  |  |
| Female gender | 0.85 | 0.78 | 0.91 |
| Age | 0.98 | 0.98* | 1.00 |
| Education | 0.82 | 1.42** | 1.72** |
| Non-Hispanic White | 1.14 | 3.31* | 2.91 |
| Non-Hispanic Black | 1.79 | 2.56 | 1.43 |
| Hispanic | 0.37 | 0.48 | 1.31 |
| Personal history of cancer | 1.19 | 2.23 | 1.87 |
| Family history of cancer | 0.82 | 1.12 | 1.37 |
| The goal of palliative care is to manage pain and other physical symptoms. |  |  |  |
| Female gender | 0.65 | 0.95 | 1.46 |
| Age | 0.97* | 0.98 | 1.01 |
| Education | 1.03 | 1.57** | 1.54* |
| Non-Hispanic White | 1.39 | 2.42 | 1.74 |
| Non-Hispanic Black | 0.39 | 2.06 | 5.29 |
| Hispanic | 0.89 | 0.43 | 0.48 |
| Personal history of cancer | 0.57 | 2.12 | 3.74 |
| Family history of cancer | 1.46 | 0.75 | 0.51 |
| The goal of palliative care is to offer social and emotional support. |  |  |  |
| Female gender | 0.83 | 0.81 | 0.98 |
| Age | 0.96** | 0.98* | 1.02 |
| Education | 0.94 | 1.39* | 1.48 |
| Non-Hispanic White | 3.75* | 3.85** | 1.03 |
| Non-Hispanic Black | 6.64* | 1.91 | 0.29 |
| Hispanic | 0.42 | 0.47 | 1.12 |
| Personal history of cancer | 1.11 | 1.48 | 1.33 |
| Family history of cancer | 3.10** | 1.21 | 0.39 |
| The goal of palliative care is to give patients more time at the end of life. |  |  |  |
| Female gender | 0.65 | 1.16 | 1.78 |
| Age | 1.01 | 0.98** | 0.97** |
| Education | 0.71** | 1.09 | 1.53** |
| Non-Hispanic White | 0.59 | 0.99 | 1.68 |
| Non-Hispanic Black | 0.55 | 1.65 | 3.01 |
| Hispanic | 1.18 | 0.48 | 0.41 |
| Personal history of cancer | 0.77 | 2.70* | 3.51** |
| Family history of cancer | 0.82 | 0.95 | 1.16 |
| Palliative care is the same as hospice care. |  |  |  |
| Female gender | 1.47 | 1.43 | 0.97 |
| Age | 1.01 | 1.00 | 0.99 |
| Education | 0.70** | 1.05 | 1.50** |
| Non-Hispanic White | 0.59 | 0.63 | 1.08 |
| Non-Hispanic Black | 1.11 | 1.11 | 1.01 |
| Hispanic | 1.26 | 0.62 | 0.49 |
| Personal history of cancer | 1.17 | 1.57 | 1.34 |
| Family history of cancer | 0.95 | 1.29 | 1.35 |
| If you accept palliative care, you must stop other treatments. |  |  |  |
| Female gender | 1.29 | 1.13 | 0.88 |
| Age | 1.01 | 1.01 | 1.00 |
| Education | 0.81* | 1.1 | 1.37** |
| Non-Hispanic White | 0.68 | 0.67 | 0.99 |
| Non-Hispanic Black | 0.85 | 0.81 | 0.95 |
| Hispanic | 1.8 | 1.04 | 0.58 |
| Personal history of cancer | 0.86 | 1.26 | 1.47 |
| Family history of cancer | 0.84 | 0.90 | 1.07 |
| **Beliefs about palliative care** |  |  |  |
| Accepting palliative care means giving up. |  |  |  |
| Female gender | 0.93 | 0.98 | 1.06 |
| Age | 1.01 | 0.97* | 0.96** |
| Education | 0.75** | 1.30* | 1.74** |
| Non-Hispanic White | 0.26** | 0.98 | 3.77* |
| Non-Hispanic Black | 0.27* | 0.46 | 1.70 |
| Hispanic | 1.65 | 0.49 | 0.30* |
| Personal history of cancer | 0.97 | 2.09 | 2.15 |
| Family history of cancer | 1.54 | 1.38 | 0.90 |
| When I think of "palliative care," I automatically think of death. |  |  |  |
| Female gender | 0.92 | 0.78 | 0.84 |
| Age | 1.00 | 0.97** | 0.97** |
| Education | 0.91 | 1.55** | 1.71** |
| Non-Hispanic White | 0.90 | 2.60 | 2.90 |
| Non-Hispanic Black | 0.69 | 1.48 | 2.15 |
| Hispanic | 0.68 | 0.46 | 0.68 |
| Personal history of cancer | 1.14 | 1.29 | 1.13 |
| Family history of cancer | 0.77 | 1.05 | 1.36 |
| It is a doctor’s obligation to inform all patients with cancer about the option of palliative care. |  |  |  |
| Female gender | 0.70 | 0.76 | 1.10 |
| Age | 1.00 | 1.01 | 1.00 |
| Education | 0.98 | 1.25* | 1.27 |
| Non-Hispanic White | 1.22 | 0.95 | 0.78 |
| Non-Hispanic Black | 0.40 | 0.80 | 2.02 |
| Hispanic | 0.28* | 0.46 | 1.64 |
| Personal history of cancer | 0.91 | 0.79 | 0.87 |
| Family history of cancer | 1.07 | 2.01 | 1.88 |

**p*<.05, ***p*<.01
